# Impact of Environmental Regulation on Regional Innovative Ability: From the Perspective of Local Government Competition

**DOI:** 10.3390/ijerph20010418

**Published:** 2022-12-27

**Authors:** Dongling Wang, Yuming Zhang, Xiaoyi Zhang

**Affiliations:** 1 School of Management, Shandong University, Jinan 250001, China; 2 School of Public Finance and Taxation, Shandong University of Finance and Economics, Jinan 250001, China

**Keywords:** environmental regulation, regional innovative ability, local government competition, dynamic spatial Durbin mode

## Abstract

We empirically investigate the relationship between environmental regulation and regional innovative ability from the perspective of government competition with a dynamic spatial model, using the 2011–2020 Chinese interprovincial panel dataset as the sample. Empirical underpinnings reveal the interconnection between environmental regulation and regional innovative ability. Further, it has been substantiated as follows: (1) environmental regulation affects regional innovative ability significantly. From the national level perspective, environmental regulation is negatively correlated with regional innovative ability. Further, vigorous environmental regulation deters regional innovative ability and produces a crowding out effect; (2) Environmental regulation has a significant spatial spillover effect on regional technological innovative ability. Meanwhile, the promulgation of environmental policy in the region will affect the innovative ability of neighboring regions; (3) It has also been concluded that there is a strategic competition among local governments in promulgation of environmental regulation, specifically in eastern and central regions which has been signified through regional regressions result. Comprehensively, the current study provides recommendations to governments for allocation of environmental policy under the aegis of regional innovation for improving regional innovative ability.

## 1. Introduction

Reforms and openness towards market economy have boosted Chinese economic and social achievements, but this extensive development is driven by production factors causing deterioration of environmental issues such as air pollution, water pollution, and land desertification [1]. Some Chinese cities are listed among the most polluted cities in the world [2]. Amid increasing concern over environmental issues, the Chinese government endeavors to improve the environment by ameliorating environmental regulation and policies while enhancing economic development quality [3,4]. Through fiscal decentralization reform and market-oriented reform, central government has gradually decentralized the authority of economic decision-making towards local governments. However, motivated by economic benefits, local governments rely on environmental regulation policy as a major tool to escalate economy and form the problem of environmental regulation competition between regions [5,6]. Environmental regulation competition refers to the competition among local governments’ environmental policies. China’s unique decentralization system and performance appraisal mechanism based on GDP growth are the main reasons for the competition of environmental regulation among local governments in China. Ma et al. [7] found that fiscal decentralization and performance evaluation mechanism in China made local governments’ environmental policies compete with each other, and environmental regulation was regarded by local governments as a game tool to compete for liquidity resources and solidify local resources and when formulating environmental policies, they would follow the example of areas with loose environmental regulation [8,9,10]. Interpretation competition of environmental regulation has formed the scale effect [11,12]. Local governments influence regional innovative performance by competing for external innovation factors and selecting environmental regulation intensity [13]. Ambec et al. [14] suggests that a race to the top in local regulation intensity will arise if the higher-level government explicitly incorporates environmental factors into the officials’ promotion assessment system or if residents force the local government to raise environmental standards by voting with their feet. Peng [15] argues that out of local protectionism, local governments compete to lower environmental standards to protect local pollution-intensive manufacturers from losing competitive advantage or attracting business from other regions, resulting in a race to the bottom in regional environmental policy. Empirically, domestic researchers have also found evidence for the race to the bottom in environmental regulation from provincial or municipal data [16,17]. Given the close relevance of environmental regulation intensity with competition for economic resources and selection of business location and its potential influence on how innovation is made in and around the region [18], in the context of a multitask principle-agent system, what are the local government environmental regulation policy which are ultimately interlinked with competitive strategies? Further, it will be quite significant to ascertain whether environmental regulation policy competition affects the innovative capability of the region itself and other regions or not? Answers to these questions will help the central government to comprehend the interactions among local government environmental policies and formulate more effective environmental policies to achieve synergistic innovation among individual regions.

Compared to the existing research, the contribution of this paper is mainly reflected in the following two points: firstly, we add a spatial weight term of environmental regulation into the spatial model to examine how adjustment to regional environmental regulation intensity potentially affects the strategic responses of governments of neighboring regions to help determine the synergy among Chinese environmental regulations; secondly, we enrich the Porter Hypothesis. We discuss the impact of environmental regulation on innovative capability from the government competition perspective, identifying the environmental regulation, competitive strategies as adopted by local governments and explore the impact of different environmental regulation competitive strategies on regional innovative ability to provide clues of realistic significance for subsequent research efforts.

We proceed as follows: Section 2 discusses the literature and theory, Section 3 explains how to build the model, Section 4 illustrates how variables are measured and descriptive statistics of variables, Section 5 encapsulates the results and discussion, and Section 6 provides recommendations and points out the shortcomings of the article.

## 2. Literature Review

Environmental regulation and regional innovation are still debatable topic within the academic community. Some researchers argue that environmental regulation affects innovative capability negatively while others believe that environmental regulation boosts it.

Regarding the relationship between environmental regulation and innovative ability, most scholars deem environmental regulation as an effective counterforce based on the Porter Hypothesis [19,20,21] enunciating that environmental regulation will make a stimulant force “fine wash” within firms to enhance innovative ability through the survival of the fittest [22]. Hamamoto [23] examines Japanese firms, witnessing that with the increase in investment expenditure on environmental pollution control, the corresponding R&D expenditure will also increase, which enhances the innovative capabilities vehemently. Liu and Yan [24] have demonstrated that environmental regulation imposes a significant impact on invention patents and utility model patents despite a lag, but its impact on design patents is insignificant. However, environmental regulation affects regional innovation differently among different regions. Shen [25] has unveiled that the impact of environmental regulation intensity on innovation is non-linear and their relationship is U-shaped. Explicitly, the impact of the enhancement of environmental regulation intensity on innovation firstly decreases and then increases, which can be explained from the perspective of path dependence of technological progress [26] which is closely interlinked with regional or corporate endowments and geographical location [27].

Under the patronage neoclassical theory, some scholars have contemplated that the amelioration of the negative externalities of the environment. Governments can also affect firm innovative capability and industrial competitiveness by increasing the production costs of the regulated firms or departments [28,29]. Hence, environmental policies can mitigate the negative external costs to the society but at high private costs [30,31,32]. Environmental regulations may impose substantial compliance costs on firms, which can reduce the firms’ financial capacities to invest in new products. Thus, environmental policy can deter firm’s productivity and innovative activities in financially constrained firms with limited internal resources and low access to external finance.

To encapsulate, some inspiring outcomes have been reported regarding the impact of environmental regulation on innovative ability. However, the extant literature has disregarded two dimensions. Firstly, due to their limited research perspectives, the researchers contemplated the environmental regulation as being given endogenously. That is, they investigated the impact of environmental regulation on regional innovative ability from a local, stationary perspective, assuming that environmental regulation policies are regionally homogeneous (within-policy). However, they neglected the correlation between policies (cross-policy) which can underestimate the impact of inter-regional environmental regulation competition on economic phenomena and lack the necessary explanatory power to the reality. Secondly, existing empirical studies mostly rely on non-spatial econometric models and methods that ignore the spatial dependencies or spatial autocorrelation inherent to spatial objects. Ultimately, it can lead to biased model assumptions or imprecise research conclusions. In this sense, it is quite necessary to explore the spatial correlation between environmental regulation and regional innovative ability through the lens of policy correlation.

Based on intergovernmental competition theory, we develop a spatial Durbin model describing the impact of environmental regulation on regional innovative ability, identifying the environmental regulation competition strategies among local governments in China with parametric symbols and measuring the spatial spillover of environmental regulation on regional innovative ability.

## 3. Model Construction

### 3.1. Spatial Econometric Model

Spatial models include spatial autoregressive model, spatial error model, and spatial Durbin model [33]. Spatial Durbin model, which considers the endogenous and exogenous variables of spatial lag, is more universal than the spatial autoregressive model and the spatial error model [34]. In fact, spatial Durbin model has superiority over the other two models. Firstly, it can distinguish between direct and indirect effects. Direct effect is the impact imposed by regional factor input variations on the region’s output; indirect effect is the impact received by the region’s output from the factor input variations of neighboring regions, usually interpreted as spatial spillover. Secondly, it analyzes total spatial effect. It can distinguish the influence of different orders, such as the first and second order, on spatial spillover, which declines with the increase in order. Thirdly, its parameters are more reliable as it incorporates spatial lag into the model while avoiding biasedness or inconsistent estimates which are associated with the endogenous nature of variables in traditional econometric models and yield more appropriate model coefficients. Henceforth, we endorsed spatial Durbin model for empirical analysis in the current study [35]. Considering the path dependency and output lag of innovation [36], we executed a dynamic spatial Durbin model (DSDM) by introducing a first-order lag term into spatial Durbin model.

We employed approaches endorsed by Gang [37] and Chen and Shu [38] to address the endogenous of intergovernmental environmental regulation competitive behaviors. Firstly, we have executed maximum likelihood estimation to acquire uniformly unbiased estimates so that the coefficients of spatial terms remain constrained by the Jacobian term in the log-likelihood function. Secondly, we chose spatial Durbin model by introducing a spatial lag term of explanatory variables which can have a correlation with missing variables while providing a solution to the endogeneity interlinking with missing variables. Thirdly, we employed panel data to control the impact of other entity (time)-dependent but not time (entity)-dependent factors for regional innovative ability and partially eliminates the endogeneity related to missing variables.

Regional features affect innovative ability [39]. Foreign investment provides monetary support for local innovation [40]. Certainly, every individual is the carrier and main performer of innovation. The degree and level of education determine how innovation is prevailed. Social development and social security expenditures in governmental expenditure is beneficial to firms to increase marginal earning rate on innovation [41]. Arguably, we incorporate foreign direct investment (FDI), human capital, and general government expenditure into the spatial econometric model as control variables to discuss how environmental regulation can influence regional innovative ability under these control variables. To investigate the spatial effect of environmental regulation on regional innovative ability, we construct a spatial Durbin model as showed in Equation (1).

(1) $$Innov_{it}=\rho \sum_{j=1}^{n}w_{ij}Innov_{it}+\lambda Er_{it}+\beta x_{it}+\eta \sum_{j=1}^{n}w_{ij}Er_{it}+w\beta x_{it}+u_{it}+v_{it}+\varepsilon_{it}$$

where: *i* represents the region and *t* represents the time; $Innov_{it}$ is the innovative ability degree of region *i* in phase *t*; $w_{ij}$ is the spatial weight matrix; $Er_{it}$ is the environmental regulation degree of region *i* in phase *t*; $x_{it}$ is the control variable affecting innovative ability degree; $u_{i}$ and $v_{i}$ are the time and spatial fixed effects; $\varepsilon_{it}$ is the error term. *ρ* is the spatial regression (lag) coefficient; *λ* is the coefficient of the direct impact of environmental regulation on the level of regional technological innovation; *η* reflects the spatial spillover effect of environmental regulation on regional technological innovative ability.

Considering the path dependency and output lag of innovation [42], namely, the potential positive or negative impact of the innovation in the previous phase on that in the next phase, a first-order lag term of local innovation has been embedded into Equation (1), resulting in a dynamic spatial Durbin model (DSDM), as showed in Equation (2).

(2) $$Innov_{it}=Innov_{it-1}+\rho \overset{n}{\underset{j=1}{\sum }}w_{ij}Innov_{jt}+\lambda Er_{it}+\beta x_{it}+\eta \overset{n}{\underset{j=1}{\sum }}w_{ij}Er_{jt}+u_{it}+v_{it}+\varepsilon_{it}\;$$

where: *i* represents the region and *t* represents the time; *ρ* is the spatial autoregressive coefficient. Further, the dependent variable “$Innov_{it}$” is the innovative ability degree of region *i* in phase *t*. Additionally, among independent variables $w_{ij}$ is the spatial weight matrix; $Innov_{jt}$ is the environmental regulation degree of region *j* in phase *t*. Moreover, $Innov_{it-1}$ is the innovation degree of region *i* in phase *t −* 1; $Innov_{it}$ is the first-order lag term of $Innov_{it}$; $Er_{it}$ is the environmental regulation degree of region *i* in phase *t*; $x_{it}$ is the control variable affecting innovative ability degree; $u_{it}$ and $v_{it}$ are the time and space fixed effects; $\varepsilon_{it}$ is the error term. ρ is the spatial regression (lag) coefficient, *λ* is the coefficient of the direct impact of environmental regulation on the level of regional technological innovation and *η* reflects the spatial spillover effect of environmental regulation on regional technological innovative ability.

### 3.2. Selection of Spatial Weight Matrix

Building a spatial weight matrix is a prerequisite to spatial econometric analysis. In a spatial model, spatial weight matrices frequently include geographic distance matrix, economic distance matrix, and economic–geographic matrix [43]. Environmental regulation and regional innovative ability are spatially correlated variables in this study. The setting of spatial weight matrix represents local governments’ selection of competitors and judgment of its own influential degree. When developing local environmental regulation according to the behaviors of a surrounding region, local governments must realize the influential degree of surrounding regions which are relevant with geographical distance. Conclusively, the closer the geographical proximity, the more likely the government’s environmental policies are to mimic each other [44]. The spillover or crowding out of innovation triggered by environmental regulation competition is also linked to economic development degree. In this regard, the developed regions have a greater economic effect spatially to less developed ones. Economic gradient and divergences are significant motivation for the regions to lower environmental regulation thresholds [45]. For this reason, we chose an economic–spatial distance matrix as the spatial weight matrix to further examine how environmental regulation spatially affects the regional innovative ability under the combination of economic and geographic distance.

Geographical matrix is calculated by the reciprocal of the linear distance between the capital cities of the two provinces, such as 1/*gd_ij_*. Economic distance is measured by the reciprocal of the economic gap between two provinces, written as $\;w_{2ij}=\frac{1}{\left|GDP_{i}-GDP_{j}\right|}$, where $GDP_{i}$ and $GDP_{j}$ are the per capita *GDP* of province *i* and province *j*; $w_{2ij}$ equals to 1 when the economic levels of the two provinces are the same *(GDP_i_ = GDP_j_)* and approximate 0 when they are largely divergent. A hybrid spatial weight matrix of geographic distance and economic distance is obtained by multiplying geographic distance matrix and economic distance matrix. It is represented as Equation (3)

*W_ij_* = 1/(*gd_ij_* × *w_*2*ij_*) (3)

We normalize Equation (3) and introduce it into Equation (2). We note that as the per capita regional *GDP* is dynamic, the spatial weight matrix we constructed is time dependent, too.

### 3.3. Determination of Local Government’s Environmental Regulation Policy Competition Strategy

Objectively, the current study investigates the impact of environmental regulation on regional innovative ability from the perspective of local government competition. Henceforth, it is necessary to identify the environmental regulation competitive strategies among local governments. Drawing inspirations from the findings of Jiang [46] and Zhao [47], in addition to the DSDM previously created (Equation (2)), we employed the direction of parameter symbols to identify different environmental regulation competition strategies, on the assumption that all these parameters are tested to be significant. It is represented as Table 1.

λ is the coefficient of *Er*, which represents the local effect of environmental regulation on regional innovative ability; *η* is the coefficient of *w*×*r*, which represents the spatial effect of environmental regulation on innovation of neighboring regions. Specifically, when λ > 0, then it indicates that there is a positive correlation between local environmental regulation and regional technological innovative ability whereas local governments adopt yardstick competition (*η* > 0). Thus, the strengthening of local environmental regulation will lead to the improvement of environmental regulation in neighboring regions, which will promote the regional technological innovation capability of neighboring regions. Further, it illustrates that there is a positive spatial spillover of environmental regulation on regional technological innovation capability. If local governments adopt differentiated competition (booster) in environmental regulation (*η* < 0), then strengthening of local environmental regulation will lead to the relaxation of environmental regulation in adjacent regions, which will ultimately weaken the level of regional technological innovation in adjacent regions (meaning that there is a negative spatial spillover of environmental regulation competition on regional technological innovative ability). Reciprocally, when *λ* < 0, then it indicates that there is a negatively correlation between local environmental regulation and regional technological innovative ability. Argumentatively, when local governments adopt differentiated competition (inhibitor) in environmental regulation (*η* > 0) then strengthening of local environmental regulation will lead to the weakening of environmental regulation in adjacent areas, thus improving the level of regional technological innovation in adjacent areas (which means that there is a positive spatial spillover of environmental regulation on regional technological innovative ability). Further, when local governments adopt the race to bottom competitive strategy in environmental regulation (*η* < 0), then relaxation of local environmental regulation will lead to more relaxation of environmental regulation in adjacent areas. Thus, reducing the innovative technological ability of neighboring regions elucidates a negative spatial spillover of environmental regulation.

## 4. Data and Variables

### 4.1. Measurement of Variables

We treat regional innovative ability as the explanatory variable, environmental regulation as the core explanatory variable. Additionally, and FDI, general government expenditure and education level are considered as control variables. These variables are measured and sourced as following.

Regional innovative ability is considered as better regional R&D ability corresponds to higher firm competitiveness in the region. Definitely, R&D ability enhances firm ability to absorb technological spillovers. Under strict environmental regulations, splendid R&D ability assists to boost firm independent innovation [48]. Following the method of Wang [49], regional innovation abilities are measured by the proportion of R&D expenditure in GPD (based on China Statistical Yearbook and China Science & Technology Yearbook data).

Environmental regulation is inspired by the findings in prior studies [50,51], environmental regulation intensity is indirectly measured by firm environmental compliance cost, i.e., the operating cost of industrial (waste water, waste gas) treatment facilities.

This study depends on the publication data from China Environmental Statistical Yearbook issues 2011 and 2020.

Control variables such as FDI is calculated by the proportion of the actual amount of FDI in GDP [52] (based on China Statistical Yearbook data). Meanwhile, education level is measured by average years of education for the labor force as described in Barro and Lee [53]. According to the length of school at different stages in China, we assume that students receive an average of 6, 9, 12, and 16 years of school education in primary school, junior middle school, senior middle school, and college and above. Thus, *per capita years of education = a*_1_ *×* 6 *+ a*_2_ *× 9 + a*_3_ *×* 12 *+ a*_4_ *×* 16, where *a_i_* is the proportion of education degree in people aged 6 and above, based on Chinese Demographic Statistical Yearbook data [54]. Further, general government expenditure is calculated by the proportion of the amount of general government expenditure to GDP (based on China Statistical Yearbook data).

### 4.2. Descriptive Statistics of Variables

Data were accumulated from 2011–2022 China Statistical Yearbook, China Science & Technology Statistical Yearbook, China Environmental Statistical Yearbook, and China Demographic Statistical Yearbook. However, Tibet is not included because complete data are currently unavailable. In real estimation, we took the log of all variables to eliminate the potential effect of heteroscedasticity. We apply maximum likelihood estimation to statistically test the spatial econometric model. From the descriptive statistics in Table 2, the maximum–minimum differences and standard deviations of regional innovative ability, environmental regulation, FDI, education level, and general government expenditure are modest. The dispersion between individuals is limited. The distribution is uniform.

## 5. Empirical Results and Discussion

### 5.1. Spatial Correlation Test

To determine the use of spatial econometric method, one needs to consider whether the main variables are spatially auto correlated. We use Moran’s I to test spatial autocorrelation, as represented by Table 3. Z-value is greater than 2.58. Moran’s I is significantly positive at 10% and its variation is modest. It shows that the observed spatial pattern cannot be randomly generated. Therefore, the null hypothesis is rejected, the spatial effect is significant. There is a stable spatial autocorrelation among historical interprovincial innovation levels.

### 5.2. Model Rationality Test

To ensure the rationality of the spatial econometric model, we perform an LM and LR test on the spatial Durbin model which has been represented by Table 4. LM test lag and Robust-LM (lag) are significant at 5%, suggesting a spatial correction in the model. The P value of both LM error and Robust-LM error is significant at 1%, suggesting a spatial lag in the selected model. This witness that our spatial Durbin model is properly formulated and well defined. The test result of both LR lag and LR error is significant at 1%, suggesting that the selected model cannot degenerate into a spatial lag model (SLM) or spatial error model (SEM).

Given the two dimensionality of panel datasets, before a panel dataset is regressed, it has to be clarified first, which type of panel regression model should be selected. Hence, fixed effect panel regression is selected via confirmation of The Housman test (the result is significant at 1%, suggesting a fixed effect). Although the log-likelihood of a time effects model is higher, the fitting degree of the model is lower than that of an entity–time fixed effects model, but the later has passed the significance test for all variables. Therefore, we assume that an entity–time fixed effects model is more rational than other models, as represented in Table 4.

### 5.3. Empirical Results from the National Sample

To avoid regression coefficient errors caused by spatial effects, we apply the partial differential estimation algorithm according to the research results of LeSage and Pace [55], and decompose the total effect into direct effect and indirect effect. We use direct effect to interpret the average impact of explanatory variables on the region and indirect effect to interpret the average impact of explanatory variables on other regions. For the purpose of this study, direct effect is defined as the impact of environmental regulation changes on the innovative ability of a region; indirect effect is defined as the impact of the environmental regulation intensity of a region on the innovative ability of other regions, i.e., spatial effect. The total effect is the sum of direct effect and indirect effect.

Empirical results of environmental regulation on regional innovative ability. From the perspective of the direct effect of environmental regulation on regional technological innovative ability, the impact factor of environmental regulation on local innovative ability is negative (−0.604) whereas it is significant at the 5% confidence level, suggesting that environmental regulation is negatively correlated with local innovative ability. Specifically, the indirect impact factor of environmental regulation is positive (0.462) and it is significant at 10%. Arguably, the result indicates a strong positive spatial spillover. Further, strict environmental regulation in one region will lead to enhanced innovation in neighboring regions. Similarly, strict environmental regulation in one region will prompt neighboring regions to lower environmental regulation standards to attract production factors to stimulate regional innovation there. The ratio of the direct effect coefficient to the indirect effect coefficient is about −1.3, which indicates that the ratio of the role of environmental regulation on the local regional technological innovative ability and the neighboring regional technological innovative ability is −1.3:1. This illustrates that if the intensity of environmental regulation in this region is increased by 1%, the local technological innovative ability will be reduced by 1.3 times, which will have a 0.8 time effect on the innovative technological ability of neighboring regions. The total effect coefficient is −0.142, which is significant at 10% (as it is equal to the sum of direct effects and indirect effects).

Empirical results of the strategy of environmental regulation policy competition: Nationally, the coefficient value of direct effect of *Er* (λ) is negative, while the coefficient value (*η*) of spatial effect is positive. Arguably, this result witness a differential competitive strategy of environmental regulation among local governments. Under a decentralized governance regime, local government decision makers have to confront both the incentives of political promotion and the pressure of the developing economy and ensuring people’s well-being. When the government of one region strictly imposes its environmental regulation system for the sake of protection of their own interests in “sibling competition”, the governments of neighboring regions will inevitably turn to a diversified environmental policy competition strategy [56]. Governments of neighboring regions try to relax environmental regulation to escalate local economic growth and local regional innovation performance in order to show their superior governing ability over their counterparts and gain better opportunities for political promotion.

Empirical results of other variables. From the empirical results in Table 5, the regression coefficient of one lag phase of innovation to current innovation is positive and is significant at 1%, suggesting a “path dependence” and significant dynamic variation of regional innovation. Innovation in the previous phase will affect the performance of current innovation. The direct effect of *FDI* on innovation is negative, and the same result has been achieved for the indirect effect. One possible explanation is that the main purpose of *FDI* is to take advantage of Chinese cheap labor forces and rich resources to make optimal profits. Its contribution to improving and diffusing regional innovative ability is quite limited. The direct effect and indirect effect of general government expenditure on innovation is positive deducing that general government expenditure helps improve regional innovative ability and has a strong spatial spillover. The direct and indirect effects of regional population education level on innovation are both insignificant. One possible explanation is that China is still weak in independent innovation. Regional innovative ability relies more on technology introduction or imitation. Average regional population education level does not make a difference to innovation.

### 5.4. Empirical Results from Regional Samples

Remarkably, due to the imbalance of economic development level and the divergence of external environment across regions, environmental regulation affects regional innovative ability differently. Further, we discuss how environmental regulation affects innovation across different regions and what environmental policy competitive strategies, which are adopted there by dividing the sample area into eastern, central, and western regions. Easter regions include Beijing, Tianjin, Hebei, Liaoning, Shanghai, Jiangsu, Zhejiang, Fujian, Shandong, Guangdong, and Hainan. Central regions include Shanxi, Jilin, Heilongjiang, Anhui, Jiangxi, Henan, Hubei, and Hunan. Western regions include Inner Mongolia, Guangxi, Chongqing, Sichuan, Guizhou, Yunnan, Shanxi, Gansu, Qinghai, Ningxia, and Xinjiang. Our analysis does not include Tibet due to unavailability of data for this region. Discussion results of regional samples are presented in Table 6, Table 7 and Table 8.

Empirical results of the direct effect of environmental regulation on regional technological innovative ability: In eastern regions, environmental regulation affects innovative ability positively (0.218) and it is significant at the 10% confidence level. In central regions, environmental regulation affects innovative ability negatively (−0.089) and it is significant at the 5% confidence level. In western regions, the impact of environmental regulation on innovation performance is insignificant. There are significant differences in the extent to which environmental regulation promotes regional technological innovative ability. According to the Porter Hypothesis, the relationship between environmental regulation and innovation resembles a U-shaped curve [57]. Logically, it offers us a rationality to trace the differences of impact of environmental regulation on innovation among eastern, central, and western regions. In eastern regions, environmental regulation affects innovation positively, possibly because these regions have crossed the inflection of the U-curve and. The incentive effect of environmental regulation on innovation mitigates the costs brought by the crowding out and boosts regional innovative ability. In central regions, environmental regulation deters innovative activity, producing a pronounced crowding out effect. This coincides with the regression result of the national sample. In western regions, the direct effect of environmental regulation on innovative ability is insignificant. One possible reason for this result is leniency among the environmental regulations. Financial subsidies and tax returns provided by the government can offset the firm’s environmental costs [58]. Hence environmental regulation does not compel the firms to make fundamental technological changes for the sake of cost or performance.

Empirical results of spatial effect of environmental regulation on regional innovative ability. In eastern regions, the spatial effect is positive (0.319) and it is significant at the 5% confidence level, suggesting that strengthened environmental regulation in one region will lead to enhanced innovation in neighboring regions. That is possibly because eastern regions have crossed the deflection of the U-curve in the Porter hypothesis. Environmental regulation stimulates innovation, increasing firms’ concern about the impact of the divergence in environmental regulation degree [59]. Continuously improved local environmental quality will attract more emerging industries and hi-tech firms, continually optimizing industrial competitiveness and capacity [60] and spurring interactive innovation in neighboring regions by way of technological spillover. In eastern regions, the ratio of the direct effect coefficient to the indirect effect coefficient is about 0.7, which indicates that the ratio of the role of environmental regulation on the local regional technological innovative ability and the neighboring regional technological innovative ability is 0.7:1. If the intensity of environmental regulation in this region is increased by 1%, the local technological innovative ability will be increased by 0.7 times, which will have 1 time effect on the innovative technological ability of neighboring regions. The total effect coefficient is 0.537, which is significant at the 10%, equal to the sum of direct effects and indirect effects. In central regions, the spatial effect is positive (0.267) and it is significant at the 1% confidence level, which suggesting that tightened environmental regulation will enhance the innovation degree of neighboring regions. This is mainly because regulation works negatively on innovative ability, producing a crowding out effect. This results in firm relocation and industrial transfer, eventually leading to the relocation of production factors to neighboring regions for reallocation [61]. In central regions, the ratio of the direct effect coefficient to the indirect effect coefficient is about 0.3, which indicates that the ratio of the role of environmental regulation on the local regional technological innovative ability and the neighboring regional technological innovative ability is 0.3:1. If the intensity of environmental regulation in this region is increased by 1%, the local technological innovative ability will be increased by 0.3 time, which will have 1 time effect on the innovative technological ability of neighboring regions. Once the intensity of environmental regulation race to bottom, it will cause a vicious circle such as the multiplier effect to the region. In western regions, the spatial effect is insignificant and there is no spatial spillover. This is because the economic base in western regions is weak and economic development there is heavily reliant on central financial subsidies.

Empirical results of competitive strategy of environmental regulation policy. In eastern regions, both the coefficients of direct effect and indirect effect is positively significant, suggesting that the intergovernmental environmental regulation policy competition in eastern regions is primarily yardstick competition. When one region strictly imposes environmental regulation policy, other regions having the similar economic development level will tend to follow in the same way which ultimately improves environmental quality. Strict regional environmental regulation will attract more hi-tech firms from higher environmental regulation standards, thus affecting the local environment positively. Talents inflow and technology diffusion will also help to elevate local innovative ability [62]. In central regions, the coefficient of direct effect is negative while the coefficient of the indirect effect is positive deducing that the intergovernmental environmental regulation policy competition in central regions is quite different. Governments in central regions tend to improve competitiveness by relaxing environmental regulation. This is mainly because central regions have a short development history and their investment in environmental projects is homogeneous. Inter-regional government competition is focused on land policies, tax policies, and environmental policies which are controllable by the government [63]. Further, it prompts governments to adopt a differential competitive strategy. Environmental regulation policy competition in western regions is undeterminable because western regions cover vast areas and the inter-regional differences are tremendous.

The coefficient of regression, one lag phase of innovation to current innovation is positive. Path dependency is verified in eastern, central, and western regions. This coincides with the regression result of the national sample, suggesting that there is a time lag in innovation and it is necessary to use a DSDM. The empirical results of *Edu* and *FDI* on regional innovative ability are consistent with the national sample. The empirical results of *Gov* on regional innovative ability in the eastern and central regions are consistent with the national sample. The empirical results of *Gov* on regional innovative ability in the western region are contrary to the national sample

### 5.5. Robustness Test

To verify the robustness of the model, we use the amount of investment in environmental governance to measure environmental regulation. Empirical results indicate no obvious change in either the coefficient symbols or significance levels of the main indexes. Changing the measuring method for main explanatory variables does not affect the relationship between environmental regulation and innovation. More details are represented by Table 9.

## 6. Conclusions and Limitations

### 6.1. Conclusions

We investigated the relationship between environmental regulation and regional innovative ability from the government competition perspective while employing a dynamic spatial model for the years 2011–2020. The Chinese interprovincial panel data set was endorsed for this empirical study. We also analyzed the strategies adopted by local governments in China for environmental policy competition. The following conclusion was encapsulated through contemplating the empirical results.

Firstly, environmental regulation affects regional innovative ability significantly, verifying the Porter Hypothesis. From the perspective of direct effects, environmental regulation is negatively correlated with regional innovative ability in the national sample. Empirical underpinnings reveal that strict environmental regulation can deter the regional innovative ability. Reasonably, environmental regulation will produce a crowding out effect which suggests that under a decentralization regime, environmental regulation confines regional innovation quality. From the results of regional regression, in eastern regions, environmental regulation boosts regional innovative ability significantly. Further, among the central regions, environmental regulation mitigates regional innovative ability. From these facts we can see a U-shaped curve between environmental regulation and regional innovation. Meanwhile, within a given interval, as environmental regulation degree increases, regional innovative ability gradually reduces. From the perspective of spatial effect, the spatial spillover of environmental regulation on regional technological innovation capacity of the national sample is positive, and the spatial effect of environmental regulation in the eastern and central regions is consistent with the national sample. Hence, Environmental regulation has a strong spatial spillover effect on the promotion of regional technological innovative ability.

Moreover, the strategic interaction of a local government environmental policy makes a great difference to the innovation in other regions. Empirical results of both samples of national and central regions have signified the negative effect of the spatial lag of local government environmental policy on regional innovative ability. This indicates that given a fixed amount of resources, regions obtaining more mobile resources through environmental policy competition can invigorate their innovative ability more quickly. Hence, the strategic interaction of environmental regulation policies affects regional innovation negatively. Under a decentralized regime, the strategic interaction of environmental policies can trigger over-competition in which governments try to sacrifice environmental quality in exchange for short-term benefits, resulting in a race to the bottom in environmental policy. In the long run, this is not only detrimental to upgrade regional innovative ability but it even curtails regional innovative ability. In eastern regions, intergovernmental environmental regulation competition is primarily yardstick competition. The positive effect of strategic interaction in environmental regulation on regional innovative ability is the most pronounced effectiveness among eastern regions. Its positive spatial spillover is conducive to inter-regional industrial enhancement and transformation there.

### 6.2. Policy Suggestions

Firstly, we suggest that the central government optimizes the local performance assessment and brings changes from GDP oriented to sustainability and ecology oriented. More attention should be paid to the coordination between economic performance and ecological performance. Ecological development, environmental protection, and cyclic economy should be incorporated into the performance assessment system. A complete environmental status assessment system should be established and involve local governments, firms, and the public to evaluate local government environmental regulation more comprehensively. Objectively and effectively adopt a multi-element, green assessment system to push these local government behaviors toward yardstick competition.

Secondly, we suggest that regional governments adapt their environmental policies to local circumstances. As ecological capacity and ecological efficiency differ from one region to another, it is important for regions to draft regional development plans and environmental regulation policies according to their own conditions. Eastern regions should take advantage of their high marketization level to optimize infrastructure and services while enhancing the ecological environment. Governments of western and central regions should play a major role in the innovation system through guiding the regions toward benign competition and reverse environmental regulation competition to yardstick competition. Additionally, the characteristics of environmental issues and the underlying economic, political and social backgrounds which can determine the effectiveness of the environmental regulation instrument, governments at all levels should take differentiated environmental regulation policies for these regions.

Thirdly, we suggest that the positive role of environmental regulation be promoted from legislation, enforcement, and supervision. Legislation promotes the basis of environmental law system. Local governments should determine the appropriate environmental regulation intensity according to local particularities, industry features and industry status which can exploit the innovation offsets as identified by the Porter hypothesis. Suggestively, utilize the counterforce of environmental regulation to push high pollution firms from end-of-pipe control toward clean production. Enforcement and supervision are the main approach to law realization. Leaders at all levels should be further educated on environmental rule of law to create a legal environment where laws are strictly enforced and effectively defended. In real practice, government approval should be curtailed. Management procedures and implementation methods should be provided for all systems. An enforcement accountability system and an assessment mechanism should be established in order to address inter-system and normalize the public supervision.

### 6.3. Limitations

Our study reached insightful conclusions, but there still exist some limitations. In the current study, we did not investigate the differences between the direct and spatial effects of different types of environmental regulation on innovation. Further, we did not contemplate whether there are differences in the spatial effect of environmental regulation on different stages of innovation. Moreover, our sample size contains a 10-year dataset to verify the relationship between environmental regulation and regional innovative ability. Future study can demonstrate longer-term dataset if the researchers want to contemplate whether the spatial relationship between environmental regulation and regional innovative ability will evolve into a U or N or a wavy curve in the long run.

## Figures and Tables

**Table 1:** Identification of environmental regulation policy competition strategy.

Coefficient	*λ* > 0	*λ* < 0
*η* > 0	Yardstick competition	Differential competition (inhibitor)
*η* < 0	Differential competition (booster)	Race to bottom

In a yardstick competition, one seeks to be stronger than others. In a race to bottom, one seeks to be weaker than others. In a differential competition, one seeks to be strong when others are weak and weak when others are strong.

**Table 2:** Descriptive statistics of variables.

Name	Symbol	Max	Min	Mean	Standard Deviation
Regional innovative ability	*In*	16.621	10.419	14.182	1.332
Environmental regulation	*Er*	0.612	0.087	0.223	0.095
FDI	*Fdi*	0.082	0.001	0.023	0.018
Education level	*Edu*	12.028	6.764	8.763	0.920
General government expenditure	*Gov*	0.612	0.087	0.223	0.095

**Table 3:** Interprovincial innovative ability Moran’s I from 2011 to 2020.

Year	Moran I	Z	Year	Moran’s I	Z
2020	0.217 **	4.86	2015	0.190 **	3.87
2019	0.216 **	4.72	2014	0.171 **	3.42
2018	0.217 **	4.86	2013	0.170 **	3.33
2017	0.218 **	4.91	2012	0.167 *	3.30
2016	0.219 **	4.94	2011	0.166 *	3.27

** *p* < 0.05, * *p* < 0.1.

**Table 4:** DSDM test results.

Variable	Fixed Effect (FE)			Random Effect (RE)
	Entity and Time Fixed	Entity Fixed	Time Fixed	
Log-Likelihood	208.476	300.224	245.538	283.419
R^2^	0.967	0.930	0.956	0.910
LM test (lag)	5.357 **			
Robust-LM (lag)	12.458 ***			
LM test (Error)	180.437 ***			
Robust–LM (Error)	187.538 ***			
LR lag	15.024 ***			
LR Error	9.857 ***			
Hausman	84.020 ***			

*** *p* < 0.01, ** *p* < 0.05,

**Table 5:** Spatial measurements for environmental regulation and regional innovative ability.

	(1)	(2)	(3)
Variables	LR_Direct	LR_Indirect	LR_Total
*In_t−_*_1_	0.327 ***	0.121	0.448
	(3.373)	(0.050)	(0.255)
*E* *r*	−0.604 **	0.462 *	−0.142 *
	(−6.430)	(1.830)	(−1.683)
*Edu*	0.088	0.140	0.228
	(0.036)	(0.069)	(0.084)
*Gov*	0.167 ***	0.215 ***	0.382 ***
	(3.628)	(3.181)	(5.489)
*FDI*	−0.899 ***	−0.980 ***	−1.879 ***
	(−6.638)	(−9.970)	(−10.700)
Observations	300	300	300
R-squared	0.996	0.996	0.996
Number of id	30	30	30

*** *p* < 0.01, ** *p* < 0.05, * *p* < 0.1; The values in () are T statistics.

**Table 6:** Estimates for eastern regions.

	(1)	(2)	(3)
Variable	LR_Direct	LR_Indirect	LR_Total
*In_t−_*_1_	0.487 ***	0.061	0.544
	(3.056)	(0.016)	(0.116)
*Er*	0.218 ***	0.319 **	0.537 ***
	(2.043)	(2.688)	(5.773)
*Edu*	0.037	0.005	0.042
	(0.028)	(0.007)	(0.078)
*Gov*	0.411 ***	0.159 *	0.570 ***
	(4.804)	(1.690)	(6.024)
*FDI*	−0.484 ***	−0.590 ***	−1.074 ***
	(−4.962)	(−6.289)	(−7.777)
Observations	110	110	110
R-squared	0.724	0.724	0.724
Number of id	11	11	11

*** *p* < 0.01, ** *p* < 0.05, * *p* < 0.1; The values in () are T statistics.

**Table 7:** Estimates for central region.

	(1)	(2)	(3)
Variable	LR_Direct	LR_Indirect	LR_Total
*In_t_*_−1_	0.327 ***	0.054	0.381
	(4.375)	(0.077)	(0.034)
*Er*	−0.089 **	0.267 ***	0.176 **
	(−2.466)	(3.170)	(2.851)
*Edu*	0.022	0.028	0.051
	(0.033)	(0.039)	(0.018)
*Gov*	0.071 **	0.381 ***	0.352 ***
	(2.224)	(4.579)	(4.431)
*FDI*	−0.067 *	−0.610 ***	−0.677 ***
	(−1.679)	(−7.172)	(−7.834)
Observations	80	80	80
R-squared	0.832	0.832	0.832
Number of id	8	8	8

*** *p* < 0.01, ** *p* < 0.05, * *p* < 0.1; The values in () are T statistics.

**Table 8:** Estimates for western region.

	(1)	(2)	(3)
Variable	LR_Direct	LR_Indirect	LR_Total
*In_t_*_−1_	0.649 ***	−0.153	0.496
	(2.101)	(−0.007)	(0.170)
*Er*	−0.366	−2.304	−2.670
	(−0.431)	(−0.629)	(−0.856)
*Edu*	0.022	0.056	0.078
	(0.050)	(0.063)	(0.047)
*Gov*	−0.243 **	0.535 **	0.292 *
	(−1.800)	(2.896)	(1.939)
*FDI*	−1.119 **	−1.410 ***	−2.530 **
	(−4.872)	(−6.217)	(−7.190)
Observations	110	110	110
R-squared	0.724	0.724	0.724
Number of id	11	11	11

*** *p* < 0.01, ** *p* < 0.05, * *p* < 0.1; The values in () are T statistics.

**Table 9:** Robustness test results for environmental regulation and innovation.

	(1)	(2)	(3)
Variables	LR_Direct	LR_Indirect	LR_Total
*In_t−_*_1_	0.318 ***	0.105	0.423
	(2.716)	(0.088)	(0.102)
*Er*	−0.302 ***	0.133 ***	−0.169 ***
	(−3.710)	(2.164)	(−2.269)
*Edu*	0.033	0.066	0.200
	(0.083)	(0.103)	(0.065)
*Gov*	0.162 ***	0.211 ***	0.373 ***
	(2.253)	(2.732)	(3.582)
*FDI*	−1.005 ***	−1.201 ***	−2.506 ***
	(−4.787)	(−7.121)	(−9.051)
Observations	300	300	300
R-squared	0.602	0.602	0.602
Number of id	30	30	30

*** *p* < 0.01, The values in () are T statistics.

## Data Availability

The data that support the findings of this study are available on request from the corresponding author. The data are not publicly available due to privacy or ethical restrictions.
